# *Mycobacterium tuberculosis* Lineage Distribution in Xinjiang and Gansu Provinces, China

**DOI:** 10.1038/s41598-017-00720-9

**Published:** 2017-04-21

**Authors:** Haixia Chen, Li He, Hairong Huang, Chengmin Shi, Xumin Ni, Guangming Dai, Liang Ma, Weimin Li

**Affiliations:** 1 Zhejiang Provincial Key Laboratory for Technology and Application of Model Organisms, School of Laboratory Medicine and Life Science, Wenzhou Medical University, Wenzhou, 325035 China; 2 National Tuberculosis Clinical Lab of China, Beijing Tuberculosis and Thoracic Tumor Research Institute; Beijing Chest Hospital, Capital Medical University, Beijing, 101149 China; 3 Academy of Mathematics and Systems Science, Chinese Academy of Sciences, Beijing, 100190 China; 4 Beijing Key Laboratory in Drug Resistance Tuberculosis Research, Beijing Chest Hospital, Capital Medical University, Beijing, 101149 China; 5 Beijing Institute of Genomics, Chinese Academy of Sciences, Beijing, 100101 China; 6 Department of Mathematics, School of Science, Beijing Jiaotong University, Beijing, 100044 China; 7 Beijing Municipal Key Laboratory of Clinical Epidemiology, School of Public Health, Capital MedicalUniversity, Beijing, 100069 China

## Abstract

*Mycobacterium tuberculosis* (*M. tuberculosis*) genotyping has dramatically improved the understanding of the epidemiology of tuberculosis (TB). In this study, 187 *M. tuberculosis* isolates from Xinjiang Uygur Autonomous Region (Xinjiang) and Gansu province in China were genotyped using large sequence polymorphisms (LSPs) and variable number tandem repeats (VNTR). Ten isolates, which represent major nodes of VNTR-based minimum spanning tree, were selected and subsequently subjected to multi-locus sequence analyses (MLSA) that include 82 genes. Based on a robust lineage assignment, we tested the association between lineages and clinical characteristics by logistic regression. There are three major lineages of *M. tuberculosis* prevalent in Xinjiang, viz. the East Asian Lineage 2 (42.1%; 56/133), the Euro-American Lineage 4 (33.1%; 44/133), and the Indian and East African Lineage 3 (24.8%; 33/133); two lineages prevalent in Gansu province, which are the Lineage 2 (87%; 47/54) and the Lineage 4 (13%; 7/54). The topological structures of the MLSA-based phylogeny support the LSP-based identification of *M. tuberculosis* lineages. The statistical results suggest an association between the Lineage 2 and the hemoptysis/bloody sputum symptom, fever in Uygur patients. The pathogenicity of the Lineage 2 remains to be further investigated.

## Introduction

Tuberculosis (TB), which is primarily caused by *Mycobacterium tuberculosis* (*M. tuberculosis*), is an immense public health concern in China and worldwide. The WHO’s 2015 global tuberculosis report indicated that 1.5 million people died from TB in 2014, ranking TB alongside HIV as a leading cause of death worldwide^1^. In 2014, 930,000 cases and 38,000 deaths related to TB were reported in China, which represents the third highest prevalence of TB worldwide^1^. According to a report by the Chinese Center for Disease Control and Prevention (CDC, China), the average bacteriological culture positivity rate was 119 per 100,000 across China^2^. However, the bacteriological culture positivity rate was higher in the Xinjiang and Gansu provinces of northwestern China, such as 433 per 100,000 in Xinjiang^3^. Thus, controlling the spread of TB in these areas should be prioritized.

Determining *M. tuberculosis* genotype is critical for designing efforts to control TB due to the impact of genotype on disease outcome, vaccine efficacy and drug resistance^4^. Current trends in genotyping suggest that in strain identification^5^, the use of large sequence polymorphisms (LSPs) or single nucleotide polymorphism (SNP) will replace mobile- or repetitive-element based markers, such as spoligotyping and variable number tandem repeats (VNTR), which suffer from problems associated with convergent evolution^6^. An analysis of the international spoligotyping database (SpolB4) has shown that while many spoligotyping patterns can be classified, and some of which are congruent with the lineage determination, such as CAS genotype belonging to Lineage 3, Beijing genotype belonging to Lineage 2 and Cameroon, Ghana, H4, Haarlem, LAM, UgandaI genotypes belonging to Lineage 4. However, some isolate patterns included in SpolB4 are either ambiguous or uninformative.

*M. tuberculosis* lineages are identified by detecting LSPs or regions of difference (RDs) that represent a series of well-characterized single-event polymorphisms (i.e., deletions). *M. tuberculosis* isolates are currently classified into six major lineages globally, including the Indian Ocean Rim (Lineage 1), the East Asian (including Beijing; Lineage 2), the Indian and East African (Lineage 3), the Euro-American (Lineage 4), the West African-1 (Lineage 5) and the West African-2 (Lineage 6)^7^. These lineages tend to exhibit a high degree of geographic restriction^7–9^. In recent decades, advances in DNA sequencing technologies have generated whole-genome sequences for *M. tuberculosis* complex (MTBC) strains from around the world^10, 11^ and produced a continually growing database of SNP-based genetic markers^11–14^. Hershberg *et al*.^15^ performed a multi-locus sequence analysis (MLSA) of 108 MTBC strains, for which they determined the coding sequences for 89 genes, corresponding to a total of ~70 thousand base pairs per strain. MLSA of these globally distributed strains of MTBC resulted in a single most parsimonious phylogenetic tree with negligible homoplasy^15^. This phylogeny was also highly congruent with their previous analyses based on large sequence polymorphisms (LSPs)^7^. To further probe the robustness of the LSP-based phylogeny, Comas^5^ re-analyzed the DNA sequence data by the neighbour-joining, maximum likelihood, and bayesian methods. All three analyses yielded identical tree topologies, which were highly congruent with their previous findings based on maximum parsimony^5^. Their results suggest that LSP markers or multi-locus sequence based method yield more robust identification of *M. tuberculosis* strain lineages as compared to markers based on mobile or repetitive genetic elements, especially spoligotyping.

Previous work has suggested that genetically distinct *M. tuberculosis* strains evoke markedly different immune responses^4^. *M. tuberculosis* strains from the Lineage 2, which are highly prevalent in East Asia and Russia, were found to elicit low-protective immune response in mice and are the most virulent. In addition, different lineages exhibit distinct pathogenicity in animal models^4^. Several clinical trials have found that the Lineage 2 was associated with relapse, treatment failure, and fever during early treatment^16^. Orgarkov *et al*.^17^ found that patients who were infected with the Lineage 2 were more likely to die of TB compared to patients infected with other strains. It is therefore important to investigate the pathogenicity of distinct lineage *M. tuberculosis*.

In this study, we used LSPs to determine the lineage of 187 *M. tuberculosis* strains isolated from Xinjiang and Gansu. These isolates are also genotyped by spoligotyping and VNTR-24. According to the minimum spanning tree based on VNTR result, representative isolates were selected and sequenced to perform multi-locus sequence analysis (MLSA) and further gained insights into the evolution of *M. tuberculosis* in Xinjiang and Gansu. To optimize the treatment regimen for different lineages, we investigated the association between distinct lineages and clinical characteristics of TB patients, including fever, cough, and relapse. Our goal is to understand the composition and distributions of the *M. tuberculosis* lineages in Xinjiang and Gansu, and to explore the association of distinct Lineages of *M. tuberculosis* with clinical characteristics of infected patients.

## Materials and Methods

### Sampling and Data Collection

We obtained 187 *M. tuberculosis* isolates from a National Survey of Drug-Resistant Tuberculosis in China in 2007 which by means of cluster-randomized sampling of tuberculosis cases in the public health system^18^. In brief, the survey was completed within 9 months and 70 clusters nationwide were selected. Of 3929 patients (85.3% of all enrolled patients) all had been tested for drug susceptibility, from which 187 *M. tuberculosis* isolates were from the northwest China. In detail, the samples consisted 69 and 64 strains from Keping Town of Aksu and Yuepuhu Town of Kashgar in the Xinjiang, respectively; 54 strains from Wushan Town, Tianshui in Gansu. Demographic and epidemiological data as well as clinical symptoms (Supplementary Table S1) were recorded for each patient. Mycobacterium genomic DNA was extracted from colonies growing on Lowenstein-Jensen media. This study was granted ethics approval by Beijing Chest Hospital.

We used the Pang’s^19^ completed spoligotype data of 187 *M. tuberculosis* strains (Supplementary Dataset 1). Compared with the spoligotype patterns from SpolB4 database, strains of the Beijing (56), CAS/CAS1_DELHI (26), Cameroon (5), Ghana (16), H4 (9), Haarlem (1), LAM (1), UgandaI (3), and unknown (16) genotype were identified in Xinjiang, and genotypes of Beijing (47), and Ghana (1), UgandaII (1), Ural (2), H4 (1) and unknown (2) (Supplementary Dataset 1) were identified in Gansu. In addition, 24-loci-MIRU-VNTR genotyping was performed according to internationally standard protocols. We screened the 187 *M. tuberculosis* strains by PCR with phylogenetically informative LSPs^9^. The RD105, pks/17 bp and RD750 deletions were used to classify isolates into Lineage 2, Lineage 4 and Lineage 3, respectively^7, 20^.

### DNA Sequencing

Strains were selected for sequencing based on two priority rules: first, to include each lineage found within the three sampling districts in the study; and second, to represent major nodes in our MIRU-VNTR-based^21^ minimum spanning trees (Supplementary Fig. S1, Supplementary Dataset 2) which were constructed by BioNumerics 5.0 (Applied Maths). A total of 10 strains representative of sampling locations and the major genetic groups found in genotyping were sequenced for 89 genes following Hershberg *et al*.^15^. PCR amplifications were carried out following the conditions and thermal profiles of Hershberg *et al*. The purified PCR products were sequenced on an ABI 3730 XI automated sequencer (Applied Biosystems). Sequencing chromatograms were visually checked using Chromas software (Technelysium Pty Ltd) for base calling errors. The sequence of 7 genes with poor quality were excluded, therefore, 82 genes with totally 60058 bp for each of our 10 representative strains were analyzed in the study.

### Phylogenetic Analysis

It has been demonstrated that phylogenetic trees reconstructed from 89 genes of 108 MTBC by utilizing distinct methods (maximum likelihood, bayesian, and maximum parsimony analyses) yielded identical topologies^5^. In order to determine the phylogenetic position of our strains,the concatenated sequence of 82 genes of our 10 representative samples together with 108 global strains used to reconstruct phylogenetic tree. Each of the sequenced genes from our representative sample was aligned with the corresponding gene of the 108 strains from Hershberg *et al*. using ClustalW in MEGA v6.0.6^22^. All aligned genes were then concatenated by SequenceMatrix v1.7.8^23^. The most suitable evolutionary model was selected by Modeltest v3.7^24^ according to the Akaike information criterion. Bayesian phylogenetic inference was carried out with the selected best fit model using MrBayes v3.2.4^25^. The Markov chain Monte Carlo (MCMC) was run for 500 million generations and sampled every 500 generations. Two independent runs, each with 4 chains running simultaneously, were conducted to check the convergence of the Markov chains. After discarding the initial 10% of the generations as burn-in, the majority rule consensus tree and its posterior probability were summarized. The maximum likelihood (ML) phylogenetic analysis was carried out using PHYMLv3.1^26^ with the same model and NNI tree topology search operation. The confidence of the ML phylogeny was assessed by performing a bootstrap procedure with 1000 replicates.

### Statistical Analysis

We investigated whether there exist associations between the type of lineages and the characteristics, including demographic/clinical factors and epidemiological status (Supplementary Table S1) of the affected patient. Multivariable logistic regression (binomial and multinomial) analyses were performed on patients groups separated by ethnicity, since there is an uneven distribution of lineages across ethnicity and regions. For binomial logistic regression, the dependent variable was Lineage 2 versus non-Lineage 2 (reference) cases, and the categorical independent variables were dummy coded in Supplementary Table S2. In order to fit a parsimonious model, a stepwise procedure, forward adding variables and backward eliminating variables according to the AIC (Akaike information criterion), were applied to select the independent variables. The significant variables (*p* value < 0.05) under the final logistic regression model were extracted and their odds ratios together with 95% confidence intervals (CIs) were calculated. The robustness of the subset of selected variables was evaluated by a bootstrap procedure (Supplementary Text S1). A multinomial logistic regression model was also conducted (Supplementary Text S2). These analyses were implemented using R software 3.2.0 (R Development Core Team).

## Results

### Lineage Identification

**Figure 1:**
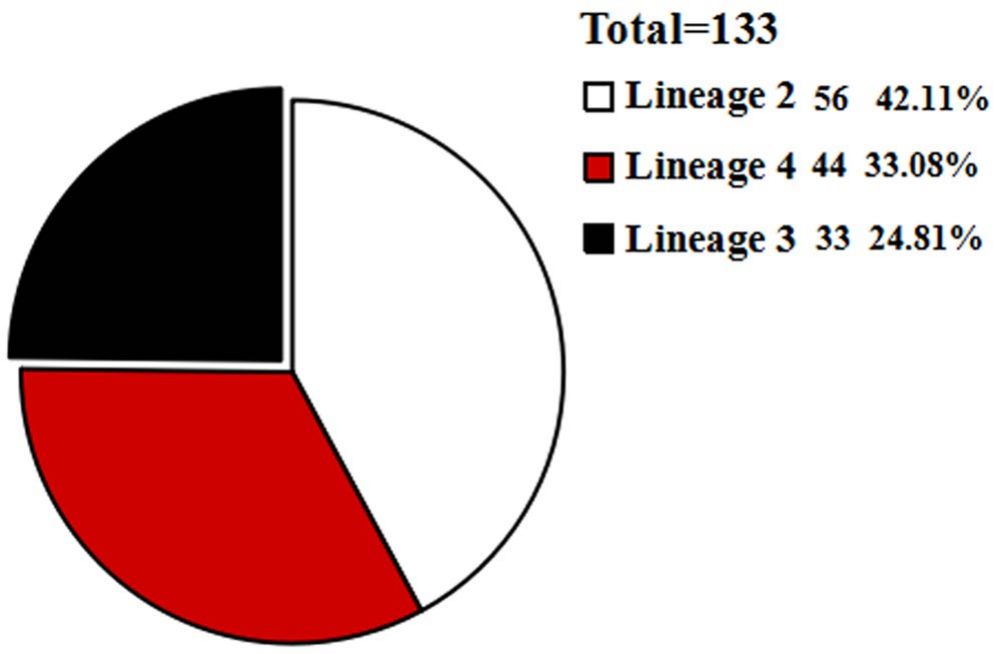
The distribution of *M. tuberculosis* strains lineage in the Xinjiang. The Lineage 2 accounts for 42.11%, the Lineage 3 and the Lineage 4 account for 24.81% and 33.08%, respectively.

**Figure 2:**
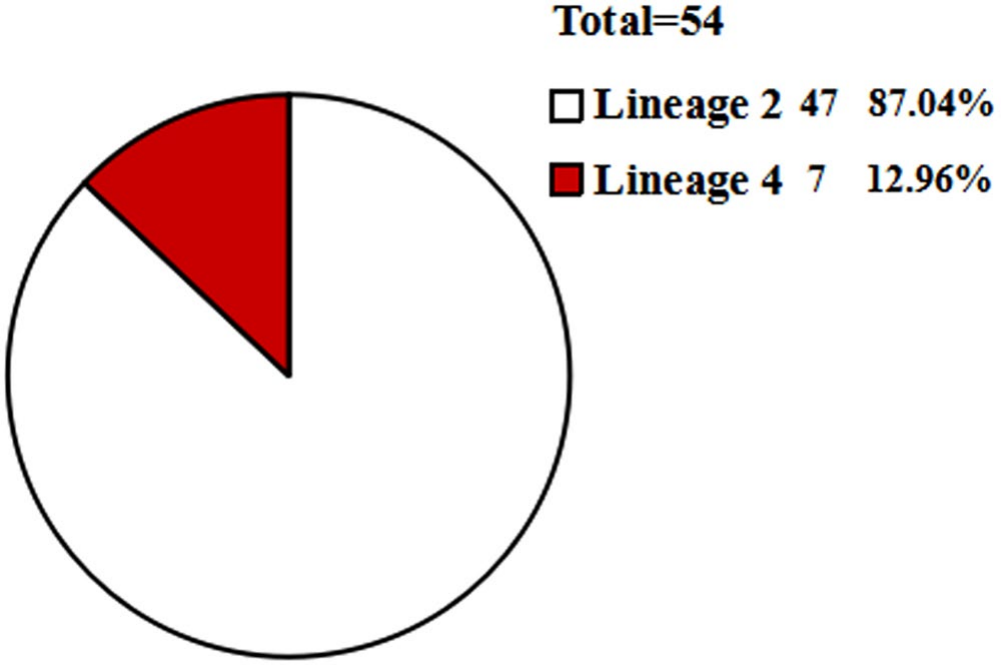
The distribution of *M. tuberculosis* strains lineage in the Gansu. The Lineage 2 accounts for 87.04% and the Lineage 4 accounts for 12.96%.

A total of 187 *M. tuberculosis* isolates from the two regions were genotyped using LSPs. Three major lineages were identified in the Xinjiang, including Lineage 2 (42.1%, 56/133), Lineage 4 (33.1%, 44/133), and Lineage 3 (24.8%, 33/133) (Fig. 1). Two lineages were identified in the Gansu, including Lineage 2 (87%, 47/54) and Lineage 4 (13%, 7/54) (Fig. 2). Although both regions exhibited a high burden of TB, their lineage distributions appeared to differ. In addition, the results of LSP confirming spoligotype show that Beijing (56) belong to Lineage 2 (56); CAS/CAS1_DELHI (25) belong to Lineage 3(25); Cameroon (5), Ghana (16), H4 (9), Haarlem (1), LAM (1), and UgandaI (3) all belong to Lineage 4 (35); in special *M.bovis* (1) belongs to Lineage 3 (1), and unknown strains (16) respectively belong to Lineage 3 (7) and Lineage 4 (9) in Xinjiang, and Beijing (47) belong to Lineage 2 (47), and Ghana (1), UgandaII (1), Ural (2), H4 (1) and unknown (2) all belong to Lineage 4 (7) in Gansu.

### The Result of Multi-locus Sequence Analysis

**Figure 3:**
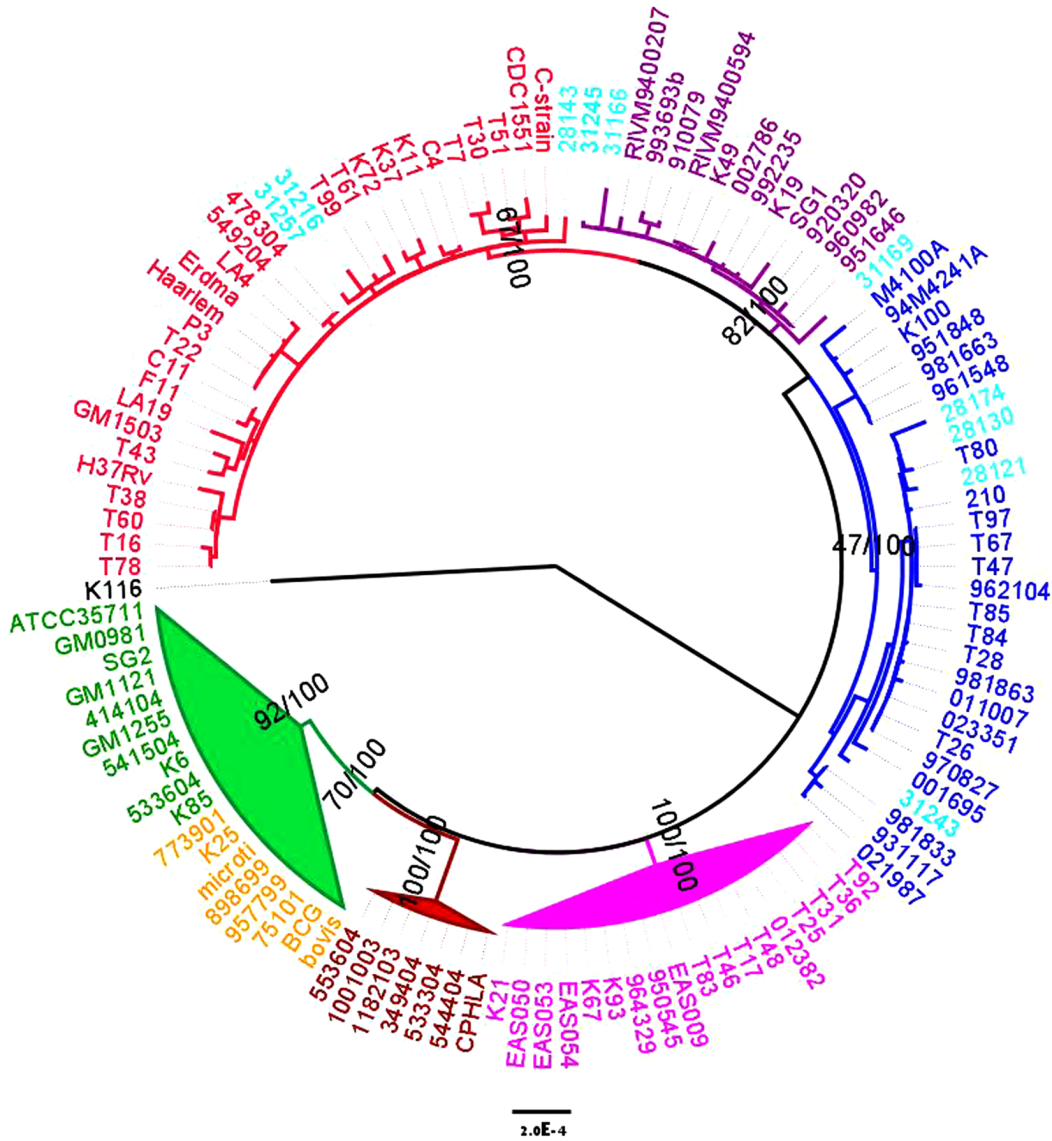
Multilocus sequence analysis phylogeny of *M. tuberculosis* complex using 82 concatenated gene sequences in 118 strains. The same topology was obtained by maximum likelihood (ML) and bayesian inference methods (BI) (see main methods for details). Values on the nodes represent clade support obtained from 1,000 bootstrap pseudo-replicates from the ML analyses and bayesian a posterior probabilities. Cyan text indicates isolates from this study. The color codes are: pink-Lineage 1, blue-Lineage 2, purple-Lineage 3, red-Lineage 4, brown-Lineage 5, green-Lineage 6 (involving the Animal Strains). The scale bar indicates the average number of nucleotide substitutions per site.

There are 82 genes, ~60 thousand base pairs, used in the phylogenetic analysis. The resulting bayesian and ML phylogenies (Fig. 3, Supplementary Fig. S2) all clustered the 108 global MTBC together with our 10 representative strains into 6 monophyletic clades, which were in agreement with Hershberg *et al*.^15^, with high bayesian posterior probabilities and moderate to high bootstrap supports for nodes (Fig. 3, Supplementary Fig. S2) that representing the six major geographical lineages. The 10 representative *M. tuberculosis* strains from our study were distributed among three distinct clades respectively representing the Lineage 2, the Lineage 3 and the Lineage 4, which is consistent with our LSP results. Therefore, the multi-locus sequence analysis appears appropriate for classifying MTBC strains into discrete lineages and reconstructing their phylogenetic relationships.

### Risk Factors

The 187 cases showed an uneven distribution of lineages between ethnicity and geographic region. All of the 54 patients from Gansu province were Han ethnicity, whereas only 3 patients in Xinjiang were of Han ethnicity. There is a much higher Lineage 2 prevalence in the samples of Han ethnicity than samples of Uygur patients (OR = 8.35). In order to reduce the influence of ethnicity and region, we applied logistic regression to patients within separate ethnic groups. For the 130 cases of Uygur ethnicity, we first applied binomial logistic analysis to Lineage 2 versus non-Lineage 2. Among the 17 independent variables, V4 (ethnicity) and V11 (region) are non-categorical variables which are not considered in the model. The variable V2 (age) is in high correlation with V3 (time in place of residence) (Pearson’s correlation 0.96). As a result, we choose to discard variable V3. The full logistic regression model was fitted based on the 14 remaining independent variables (Supplementary Table S1). Results showed that only two variables were significant, which are pulmonary tuberculosis in family members (Yes/No) and fever (Yes/No) (Supplementary Table S3). In order to refine the model, we adopted a stepwise procedure according to AIC. We also performed an ANOVA test between the full model and the final model (*p* value = 0.8774), which showed that the two models performed equivalently. The final model was constituted of five predictor variables, where fever (yes) (V13) and hemoptysis/bloody sputum symptoms (yes) (V14) were significant (Supplementary Table S4). These two variables are also relatively robust by bootstrap approach (Supplementary Table S5). The odds ratios provided in Table 1 indicate that Lineage 2 in Uygur ethnicity is more likely to be associated with fever (adjusted OR = 4.19) and hemoptysis/bloody sputum (adjusted OR = 3.36). We also did a Chi-square test for lineages and fever (Yes/No), where the *p* value (0.005183) which again indicates the correlation. The result of multinomial logistic regression for Uygur patients also found fever (yes) as significant (*p* value < 0.05) variable (Supplementary Text S2). For the Han ethnicity, however, the logistic regression model was over-fitted and may be due to the small sample of non-Lineage 2 strains. The odds ratio calculated for hemoptysis/bloody sputum symptoms (yes) in Lineage 2 and non-Lineage 2 of Han patients is about 1.452. In summary, the above analysis suggest that Lineage 2, especially in Uygur ethnicity patients, showed considerable correlation with fever and hemoptysis/bloody sputum symptoms.

**Table 1:** The odds ratios of variables associated with Lineage 2 in Uygur patients.

Variables^*^	OR^†^	95%CI	P value
Fever (yes)	4.1948	1.7669–10.8367	0.00177
Hemoptysis/bloody sputum (yes)	3.3625	1.0829–11.3966	0.04079

^†^Denotes adjusted OR in logistic regression.

## Discussion

Xinjiang is located near the northwest border of China and neighbors eight countries, including India, Russia, Mongolia, Pakistan, Kyrgyzstan and Afghanistan. Thus, Xinjiang is an important transportation hub for immigration, travel and trade between China and other countries in Central Asia. Gansu is a corridor that connects the western provinces, including Xinjiang, Qinghai and Tibet, with inland China. Although the same strategies for preventing and controlling TB have been deployed throughout China, the TB morbidity rate in Xinjiang and Gansu is much higher than the national average^2^. Thus, understanding the features of the molecular epidemic distribution of different *M. tuberculosis* lineages in the two provinces is vital to the prevention and control of TB.

We used large sequence polymorphisms (LSPs) as markers to genotype all *M. tuberculosis* strains circulating in Xinjiang and Gansu. Of course, most spoligotype patterns are congruent with the lineage determination, but some unknown patterns still need be confirmed by LSP. In Gansu, two unknown spoligotype strains belonged to the Lineage 4. Then the proportions of the Lineage 2 and the Lineage 4 were 87% (47/54) and 13% (7/54), respectively. In Xinjiang, sixteen unknown spoligotype strains respectively belong to the Lineage 3 (7) and the Lineage 4 (9). Therefore the proportions of the Lineage 2, the Lineage 3 and the Lineage 4 were 42.1% (56/133), 24.8% (33/133) and 33.1% (44/133), respectively. Because the LSP method was published 16 years ago, we should use new methods with greater discrimination to confirm LSP results. In this study we evaluated 10 representative strains with an MLSA assay, which is a robust means to classify MTBC strains into discrete lineages. This method identified the 10 *M. tuberculosis* strains in Xinjiang and Gansu as belonging to the Lineage 2 (4), the Lineage 3 (3) and the Lineage 4 (3) (Fig. 3), in agreement with our LSP classification. The agreement of two independent methods indicates that 56 out of 133 samples (42.1%) were the Lineage 2 in Xinjiang, a markedly lower proportion than the average across China^27^. In addition, there were 44 strains (33.1%) and 7 strains (13%) respectively in Xinjiang and Gansu from the Lineage 4, which were reported in the Sichuan Basin of China in our previous study^20^. Interestingly, *M. tuberculosis* the Lineage 3 was identified in Xinjiang and accounted for 24.8% (33/133) of the strains in this study. Dong *et al*.^28^ first identified CAS spoligotype *M. tuberculosis* in Xinjiang. Based on the theory of CAS spoligotype belonging to *M. tuberculosis* the Lineage 3, Wan *et al*.^29^ found nine strains from Xinjiang showed a typical lineage 3 (CAS) pattern and clustered by VNTR typing with Lineage 3 CAS-Delhi isolates from other geographical origins. Zhang *et al*.^30^ complemented whole genomic sequence of 161 *M. tuberculosis* strains from china, including one Lineage 3 from Xinjiang. In a word, we not only identified Lineage 3 using two phylogenetic markers, but also showed its proportion of the samples in two clusters in Xinjiang.

A study by Newton^31^ suggested that *M. tuberculosis* the Lineage 3 is capable of evading the immune response, which contributes to the persistence and potential for outbreaks of this lineage among human populations^31^. Similar to *M. tuberculosis* the Lineage 2 (i.e., Beijing strains), the Lineage 3 presented a relatively slow growth rate and a reduced ability to induce pro-inflammatory factors, permitting evasion of host immune responses^32^. In our samples, there are 33 Uygur ethnicity patients infected with the Lineage 3, whereas no Han ethnicity cases were found. This implies an association between Lineage 3 and the Uygur ethnicity, which support the ethnic-lineage correlation as suggested by Pareek, M. *et al*.^33^. However, due to a small number of cases caused by *M. tuberculosis* Lineage 3 and Lineage 4 in Han patients, it is difficult to evaluate the causes of the observed association between ethnicity and lineages in this study.

The Lineage 2 *M. tuberculosis* (i.e., Beijing family) has a high prevalence in China^27, 34^. Yang, C. *et al*.^34^ reported that Beijing strains of *M. tuberculosis* were significantly associated with genotypic clustering and younger age, but were not associated with drug resistance. Many studies suggested that the Lineage 2 is more virulent than other lineages^4, 17, 35^. Several studies have shown that *M. tuberculosis* the Lineage 2 is associated with clinical symptoms in patients. In Indonesia^36^ patients infected with strains of the Beijing genotype were more likely to develop fever regardless of disease severity during the first stage of treatment. However, the studies from Singapore and Russia reported a lower occurrence of fever and night sweats among patients infected with the Lineage 2^37, 38^. From a socioeconomic perspective, factors such as living conditions, nutrition and sanitation among the northwest China could increase the risk of infection. In our study, the proportion of patients affected by the Lineage 2 were 87.0% and 42.1% in Gansu and Xinjiang provinces, respectively. The logistic regression results revealed that *M. tuberculosis* the Lineage 2 in Uygur patients was correlated with fever (adjusted OR = 4.1948, 95% CI (1.7669–10.8367), *p* value = 0.00177) and hemoptysis/bloody sputum symptom (adjusted OR = 3.3625, 95% CI (1.0829–11.3966), *p* value = 0.04079). For Han patients affected with the Lineage 2, the odds of having hemoptysis/bloody sputum symptoms is 1.452 times larger than the odds for non-Lineage 2 affected patients. This suggested that patient infected with the Lineage 2 in our samples appear to be associated with fever, hemoptysis/bloody sputum symptom. These findings may help to optimize the TB therapeutics strategy based on strain lineage.

In summary, we identified *M. tuberculosis* strains from the Lineage 2, 3 and 4 in the Xinjiang and the Lineage 2 and 4 in Gansu using LSPs and VNTR. Ten isolates were selected, which represent major nodes of VNTR-based minimum spanning tree, and subsequently analyzed by MLSA. Our results indicated that MLSA-based phylogenies can be used to classify *M. tuberculosis* and provided preliminary insights into the evolution of the bacterium. Statistical analysis revealed that Lineage 2 was associated with fever and hemoptysis/bloody sputum in Uygur patients. In Han patients, there is a higher odds of hemoptysis/bloody sputum symptom in those affected by Lineage 2. These suggested that the pathogenicity of the Lineage 2 should be noted in TB patients. This study provided a basis for formulating new strategies for the control and prevention of TB in Northwest China and Central Asian countries.

## Electronic supplementary material

Mycobacterium tuberculosis Lineage Distribution in Xinjiang and Gansu Provinces, China

Dataset 1

Dataset 2
