# Supplementary material for: Mycobacterium tuberculosis Lineage Distribution in Xinjiang and Gansu Provinces, China
Source: Sci Rep. 2017 Apr 21;7:1068. doi: 10.1038/s41598-017-00720-9 (PMC5430859; doi:10.1038/s41598-017-00720-9)
Supplement: Supplementary file 1 — Mycobacterium tuberculosis Lineage Distribution in Xinjiang and Gansu Provinces, China [file 41598_2017_720_MOESM1_ESM.pdf]

Mycobacterium tuberculosis Lineage Distribution in Xinjiang and Gansu Provinces, China

Haixia Chen#[1,2], Li He#[3], Hairong Huang#[2,4], Chengmin Shi[5], Xumin Ni[6], Guangming Dai[2,4], Liang Ma*[5], Weimin Li*[2,4]

[1] Zhejiang Provincial Key Laboratory for Technology and Application of Model Organisms, School of Laboratory Medicine and Life Science, Wenzhou Medical University, Wenzhou, 325035, China

[2] National Tuberculosis Clinical Lab of China, Beijing Tuberculosis and Thoracic Tumor Research Institute; Beijing Chest Hospital, Capital Medical University, Beijing, 101149, China

[3] Academy of Mathematics and Systems Science, Chinese Academy of Sciences, Beijing, 100190, China

[4] Beijing Key Laboratory in Drug Resistance Tuberculosis Research, Beijing Chest Hospital, Capital Medical University, Beijing, 101149, China

[5] Beijing Institute of Genomics, Chinese Academy of Sciences, Beijing, 100101, China

[6] Department of Mathematics, School of Science, Beijing Jiaotong University, Beijing, 100044, China

# These authors contributed equally to this work.

*Corresponding author

E-mail: mal@big.ac.cn (L.M.) and lwm_18@aliyun.com (W.L.)

Supplementary Text S1. The stepwise regression.

In the statistical analysis, we employed a stepwise procedure (forward and backward) to refine the full logistic regression model which constitutes of 14 predictor (independent) variables. It is applied by adding or eliminating independent variables according to AIC (Akaike Information Criterion). A bootstrap procedure was conducted to assess the robustness of our approach in selecting significant variables. Totally 1000 sets of bootstrapping pseudo-samples were generated, where each set was produced by resampling the original data set with replacement (with the same size of the original data set). Following the above stepwise procedure, logistic regression models were fitted over each pseudo-samples. The number of times of significance for each variable out of 1000 bootstrapped models are shown in Table S7.

Supplementary Text S2. Multinomial logistic regression model.

In addition to the binomial logistic regression model, we also performed a multinomial logistic regression model on the patients of Uyghur. The dependent variable has three levels which respectively represent the Lineage 2 (Beijing strains), the lineage 3 (CAS strains) and the Lineage 4 (Euro-American strains). We took the Lineage 2 as the reference level and model the regression based on the same 14 predictors as in the binomial case. A similar stepwise procedure was conducted with V13 and V14 kept in the resulting final model. The variable fever (yes) (V13) was significant with p-value < 0.05.

Supplementary Figure S1

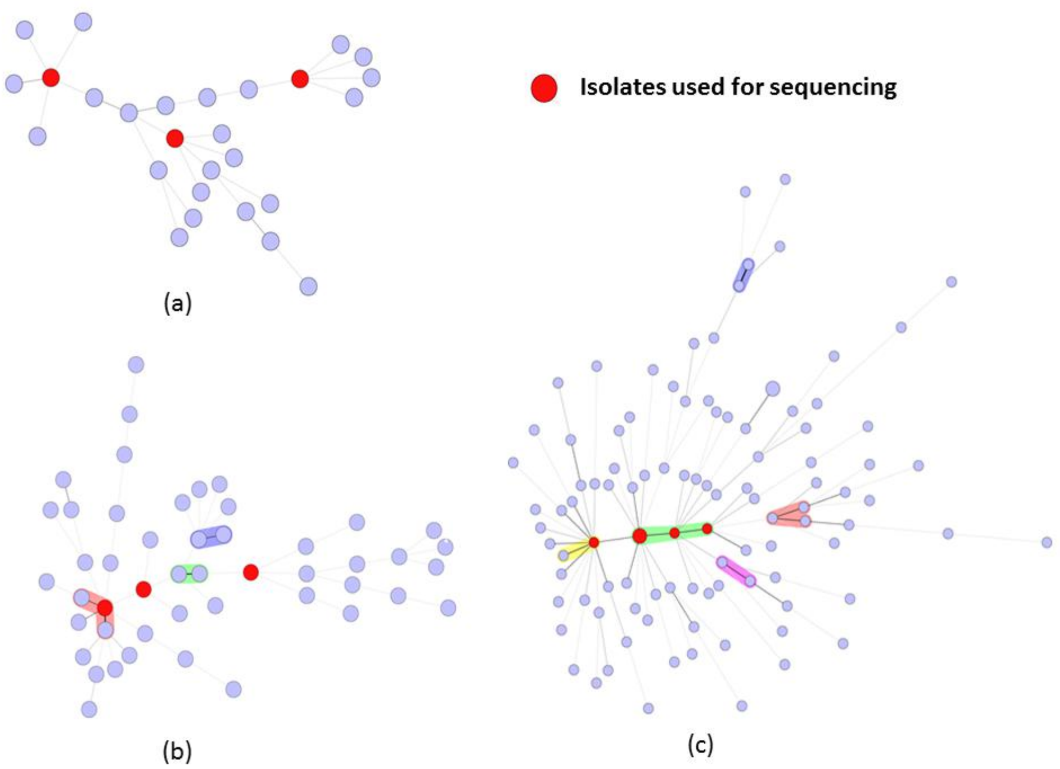

Fig. S1. The VNTR-based minimum spanning trees. Nodes in red were the selected representative strains which subsequently used for multi-locus sequence analysis. The 24-VNTR result of *M. tuberculosis* in the study was shown in Supplementary Table S3. A total 177 strains of 24-VNTR results were contructed minimum spanning trees by BioNumerics 5.0, 10 stains of 24-VNTR results were excluded becuase of missing PCR products from over 8 VNTR locis.

(a) Minimum spanning tree of the Lineage 3;

(b) Minimum spanning tree of the Lineage 4;

(c) Minimum spanning tree of the Lineage 2.

Supplementary Figure S2

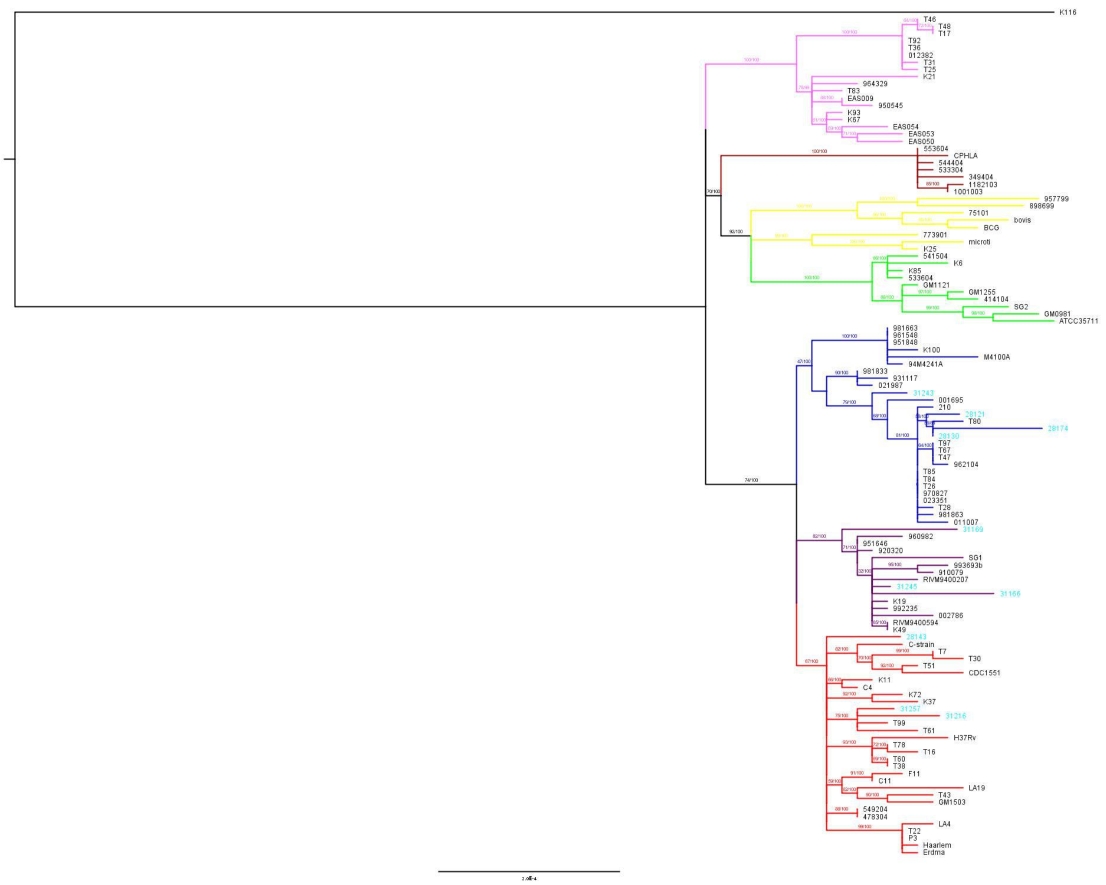

Fig. S2. Multilocus sequence analysis phylogeny of *M. tuberculosis* complex using 82 concatenated gene sequences in 118 strains. The same topology was obtained by maximum likelihood and Bayesian inference methods (see main methods for details). Values on the nodes represent percentage of clade support as obtained from 1,000 bootstrap pseudo-replicates for the ML analyses and Bayesian a posteriori. Cyan text indicates the isolates from this study. The correspondences are: pink-Lineage 1, blue-Lineage 2, purple-Lineage 3, red-Lineage 4, brown-Lineage 5, green-Lineage 6 (involving orange-the Animal Strains).. The scale bar indicates the average number of nucleotide substitutions per site.

Supplementary Table S1.

variable	demography, epidemiological and clinical symptoms
V1*	gender
V2	age
V3	time in place of residence
V4*	ethnicity
V5*	occupation
V6*	Degree of Education
V7*	income
V8	family population number
V9*	pulmonary tuberculosis in family members(Yes/No)
V10*	tuberculosis in the neighborhood, colleagues and classmates(Yes/No)
V11*	region
V12*	cough(Yes/No)
V13*	fever(Yes/No)
V14*	hemoptysis/bloody sputum symptoms(Yes/No)
V15*	thoracodynia(Yes/No)
V16*	other symptoms in this treatment
V17*	diagnosed as tuberculosis before the treatment(Yes/No)

Table S1. The detailed data of demography and epidemiological as well as clinical symptoms. Variables marked with asterisk (*) are classified in Table S4.

Supplementary Table S2.

	Value (Number)			
V1	0 male(60)	1 female(70)		
V4	0 Uyghur(130)	1 Han		
V5	0 farmers(121)	1not farmers(9)		
V6	0 illiterate/semiliterate (54)	1 primary school(40)	2 junior high school(28)	3 high school or above(8)
V7	0 <2500(19)	1 2500-4200(38)	2 4200-20000(57)	3 >20000(14)
V9	0 yes(37)	1 no(93)		
V10	0 yes(17)	1 no(107)	2 uncertain answers(5)	
V11	0 Xinjiang(Aksu/ Kashgar)(130)		1 Gansu	
V12	0 no(1)	1 yes(10)	2 long time(119)	
V13	0 no(61)	1 yes(69)		
V14	0 no(112)	1 yes(18)		
V15	0 no(42)	1 yes(88)		
V16	0 no(104)	1 yes(26)		
V17	0 yes(74)	1 no(56)		

Table S2. The numbers of Uygur patients in each categorical variables. The variables V9, V10 and V17 have different coding. V7 and V10 have missing data denoted by -1 which may influence the dummy code in logistic regression model.

Supplementary Table S3.

	Estimate	Std. Error	Z value	Pr(>|z|)
Intercept	-2.949e+01	2.058e+03	-0.014	0.98857
V1(1)	-4.522e-01	4.511e-01	-1.002	0.31621
V2	3.207e-03	1.469e-02	0.218	0.82719
V5(1)	3.312e-01	9.396e-01	0.353	0.72446
V6(1)	6.545e-01	5.476e-01	1.195	0.23198
V6(2)	4.111e-01	7.438e-01	0.553	0.58045
V6(3)	-2.140e-02	1.124e+00	-0.019	0.98481
V7(0)	1.997e-01	1.980e+00	0.101	0.91964
V7(1)	-7.194e-01	1.899e+00	-0.379	0.70481
V7(2)	-7.872e-01	1.969e+00	-0.400	0.68935
V7(3)	1.373e-01	2.102e+00	0.065	0.94790
V8	2.152e-01	1.342e-01	1.604	0.10870
V9(1)	-1.002e+00	4.949e-01	-2.025	0.04285*
V10(0)	1.320e+01	1.455e+03	0.009	0.99276
V10(1)	1.380e+01	1.455e+03	0.009	0.99243
V10(2)	1.197e+01	1.455e+03	0.008	0.99344
V12(1)	1.473e+01	1.455e+03	0.010	0.99193
V12(2)	1.465e+01	1.455e+03	0.010	0.99197
V13(1)	1.460e+00	5.455e-01	2.677	0.00743**
V14(1)	1.228e+00	6.573e-01	1.869	0.06168.
V15(1)	-4.900e-01	5.544e-01	-0.884	0.37671
V16(1)	1.227e+00	7.090e-01	1.731	0.08339.
V17(1)	1.266e-01	4.625e-01	0.274	0.78425
AIC	188.5			

Table S3. The full logistic regression model.

Lineage~V1+V2+V5+V6+V7+V8+V9+V10+V12+V13+V14+V15+V16+V17. The Lineage 2 and non-Lineage 2 are coded as 1 and 0, respectively. The variables V7 and V10 are dummy coded differently due to the missing data. Signif.codes: ~0 '***' 0.001 '**' 0.01 '*' 0.05 '.' 0.1 ' ' 1.

Supplementary Table S4.

	Estimate	Std. Error	Z value	Pr(>|z|)
Intercept	-1.66848	0.62258	-2.680	0.00736**
V8	0.18647	0.09784	1.906	0.05668.
V9(1)	-0.79671	0.43449	-1.834	0.06670.
V13(1)	1.43384	0.45871	3.126	0.00177**
V14(1)	1.21268	0.59280	2.046	0.04079*
V16(1)	0.86721	0.54686	1.586	0.11279
AIC	165.08			

Table S4. The final logistic regression model including coefficients and significance. Lineage~V8+V9+V13+V14+V16. V8: family population number V13(1): fever(Yes) V16(1): other symptoms in this treatment V14(1): hemoptysis/ bloody sputum symptoms(Yes) V9(1): pulmonary tuberculosis in family members(No).

Supplementary Table S5.

variable	number	variable	number
V13(1)	772	V17(1)	104
V9(1)	548	V2	100
V14(1)	518	V6(2)	97
V8	415	V6(3)	87
V16(1)	400	V10(1)	49
V1(1)	250	V7(1)	41
V15(1)	234	V7(2)	40
V6(1)	212	V12(2)	37
V5(1)	110	V7(3)	30

Table S5. The bootstrap results. The significant variables for each selected model are recorded. Out of the 1000 bootstrapped models, V13(1) was significant in 772 of times, and V14(1) was significant in 518 of times. Therefore, we think that the stepwise regression used in our analysis was robust.

Note *Dummy coding

Before fitting the logistic model, each categorical variable is recoded to a set of binary (zeros and ones) dummy variable. For variable with k categories, k-1 dummy variables are needed, which mapping to the categories start from the second. Each dummy variable codes 1 if the observation fall into the corresponding category and 0 otherwise. For example, in a dichotomous variable V1, male codes 0 and female codes 1. A multi-categorical variable V6 will be coded as follows:

Degree of Education	V6(1)	V6(2)	V6(3)
0: illiterate/semiliterate	0	0	0
1: primary school	1	0	0
2: junior school	0	1	0
3: High school or above	0	0	1

Supplementary Dataset 1. Spoligtype data of 187 *M. tuberculosis* strains in the study.

Supplementary Dataset 2. The 24-VNTR results of 177 *M. tuberculosis* strains in the study. 10 stains of 24-VNTR results were excluded becuase of missing PCR products from over 8 VNTR locis.
